# *Aedes* mosquito species in western Saudi Arabia

**DOI:** 10.1093/jis/14.1.69

**Published:** 2014-01-01

**Authors:** Masroor Alikhan, Khalid Al Ghamdi, Jazem Abdullah Mahyoub

**Affiliations:** 1 Insect laboratory, Al Amana, Bariman, Jeddah, Saudi Arabia; 2 Dept. of Biological sciences, King Abdulaziz University, Jeddah, Saudia Arabia

**Keywords:** *Aedes aegypti*, *Aedes caspius*, *Aedes vexans arbiensis*, *Aedes vittatus*

## Abstract

The *Aedes* Meigen (Diptera: Culicidae) mosquito species populations in the western region of Saudi Arabia, especially in and around Jeddah, are increasing, therefore increasing susceptibility of humans to the dengue virus. An extensive survey was carried out for one year, and four species were identified with the help of different pictorial keys available. The identification was based on morphological characteristics of adult female *Aedes* mosquitoes.

## Introduction

Several mosquito species are of medical importance, but *Aedes* Meigen (Diptera: Culicidae) species are becoming the most important from a medical point of view all over the world. *Aedes* species (especially *Aedes aegypti and Aedes albopictus* ) are vectors of arbo-viruses that infect various vertebrates, including humans. The most common arbo- viruses spread by *Aedes* species and infecting humans are the dengue fever virus, yellow fever virus, and chikungunya virus. The *Aedes* species of Saudi Arabia have been studied by several people, such as Mattingly and Knight (1956) , Buttiker (1979, 1981) , Wills et al (1985) , Abdullah and Merdan (1995) , Al Zahrani (2001), Jupp et al (2002) , Godsey Jr. et al (2003) , Al Kherji (2005), Azzam (2006), and Al Ahmad et al (2011) .

Mattingly and Knight (1956) identified three species from the southwest only, namely *Aedes aegypti, Aedes arabiensis* and *Aedes caspius.*Buttiker (1979, 1981) did not reported any *Aedes* species from this region. Wills et al. (1985) recorded *Aedes caspius* from the eastern part of the country. Abdullah and Merdan (1995) collected only one species, *Aedes caspius* , from the southwestern region, excluding Jeddah. Al Zaharani (2001) reported only *Aedes vittatus* from the southern region. Jupp et al (2002) mentioned four species from Jizan (southern part), *Aedes vexans arabiensis, Aedes vittatus, Aedes caspius* and *Aedes caballus* . Godsy Jr.et al. (2003) reported *Aedes unilineatus* from Makkah region. Al Kherji (2005) only collected *Aedes caspius* from Riyadh. Azzam (2006), Al Ahmad et al (2011) , and Mahyoub (2011) reported *Aedes caspius* and *Aedes aegypti* from Jeddah.

There is no authentic record of *Aedes* mosquito fauna in the western region of Saudi Arabia. In an effort to better understand the fauna, we performed a comprehensive study of the aedine mosquitoes of the western region. The present work is the result of intensive surveillance for almost one year, from January 2010 to December 2010. The study revealed the presence of four species of *Aedes* in this region. Some of them have been reported in earlier studies from different parts of Saudi Arabia.

## Materials and Methods

*Aedes* species bite during the day and sometimes early night, and dawn and dusk are the peak biting times (WHO 2008). A thorough sampling was carried out by installing the light traps in various habitats in the western region of the country. Black Hole light traps (Rubicon, Inc., ( www.btglobal.co.kr ) were used to attract the adult mosquitoes during the entire period of surveillance. Powered aspira- tors and flash torches were also used to collect mosquitoes from their different resting places.

**Figure 1.**
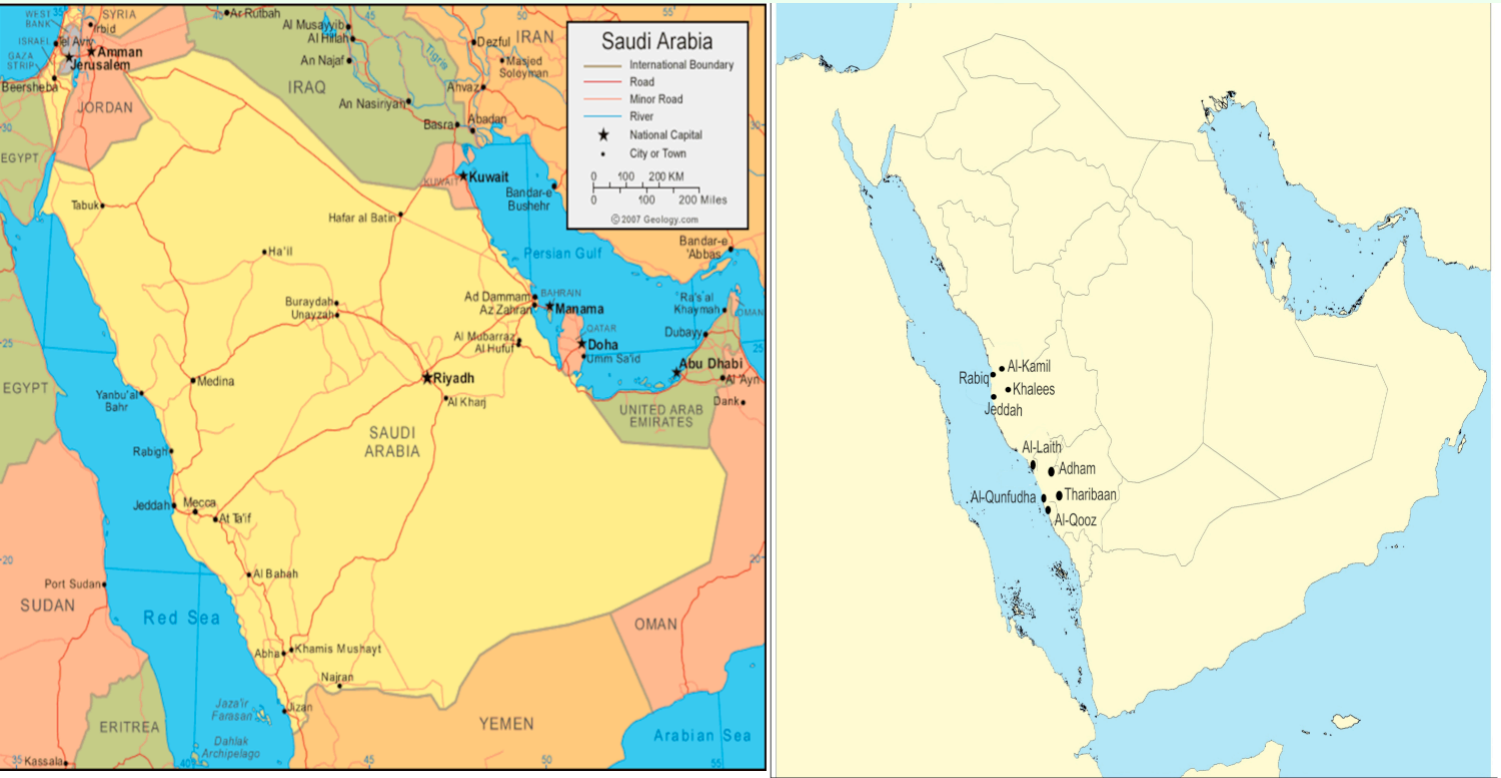
Maps of Saudi Arabia showing the distribution of *Aedes* species in the western region. High quality figures are available online.

Fourteen traps were installed in the city of Jeddah. The other towns selected for the surveillance were Al-Qunfudha, Al Qooz, Al Laith, Adham, Tharibaan, Khalees, Rabiq, and Al Kamil, and two traps were set in each city ( Figure 1 ). Live adult mosquitoes were brought to the laboratory for identification. After keeping them for 25 minutes in the deep freezer for immobilization, the samples were thoroughly searched for *Aedes* species, which were sorted out species wise and sex wise to determine the male:female ratio. Identification was done on the basis of adult female morphological characters with the help of different standard taxonomic keys and catalogues ( Barraud 1934 ; Knight and Mattingly 1956 , 1971; Knight and Stone 1977 ; Harbach and Knight 1980 , 1981; Reinert 2000 ; Rueda 2004 ; Azzam 2006).

## Results and Discussion

In the present study, four species of *Aedes* were identified from the western region of Saudi Arabia. *Aedes (Stegomyia) aegypti* (L.) was most commonly collected from almost all the locations of Jeddah and other towns. *Aedes (Ochlerotatus) caspius* (Pallas) was restricted in the regions that are close to sea. The highest number of *Aedes (Ochlerotatus) caspius* were collected from Bariman and South of Jeddah, and a few specimens were also collected from Rabiq and Al Qooz. *Aedes (Ochlerotatus) vexans* var. *arabiensis* (Meigen) was mainly concentrated in Al-Qunfudha and Al-Qooz, but a few specimens were also collected from Jeddah and other areas. *Aedes vittatus* Bigot was abundantly found in Al- Qooz and Al-Qunfudha. From the city of Jeddah, two female specimens of *Ae. vittatus* were collected from Um-Salam region only ( Table 1 ).

**Table 1.**
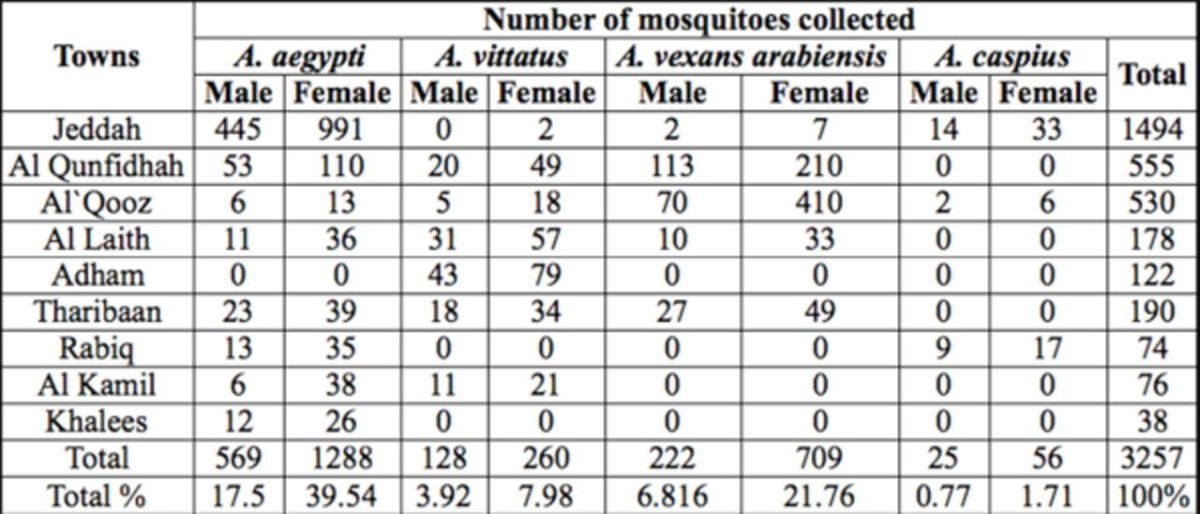
*Aedes* mosquito species of Western Saudi Arabia, 2010–2011.

The most abundant species recorded in the western region was *Ae. aegypti* (57%), followed by *Aedes vexans* (28.576%), *Ae. vittatus* (11.90%), and *Ae. caspius* (2.477%). *Aedes vittatus* and *Ae. vexans arabiensis* have not been reported previously from Jeddah city..

Correct identification of *Aedes* mosquitoes is necessary for effective control of Dengue fever and other arbo-viral diseases prevalent in this region. A simple guide with the diagnostic features is prepared to identify the *Aedes* mosquitoes of Western region of Saudi Arabia.

### *Aedes (Stegomyia) aegypti* : ( Plate I )

**Plate I.**
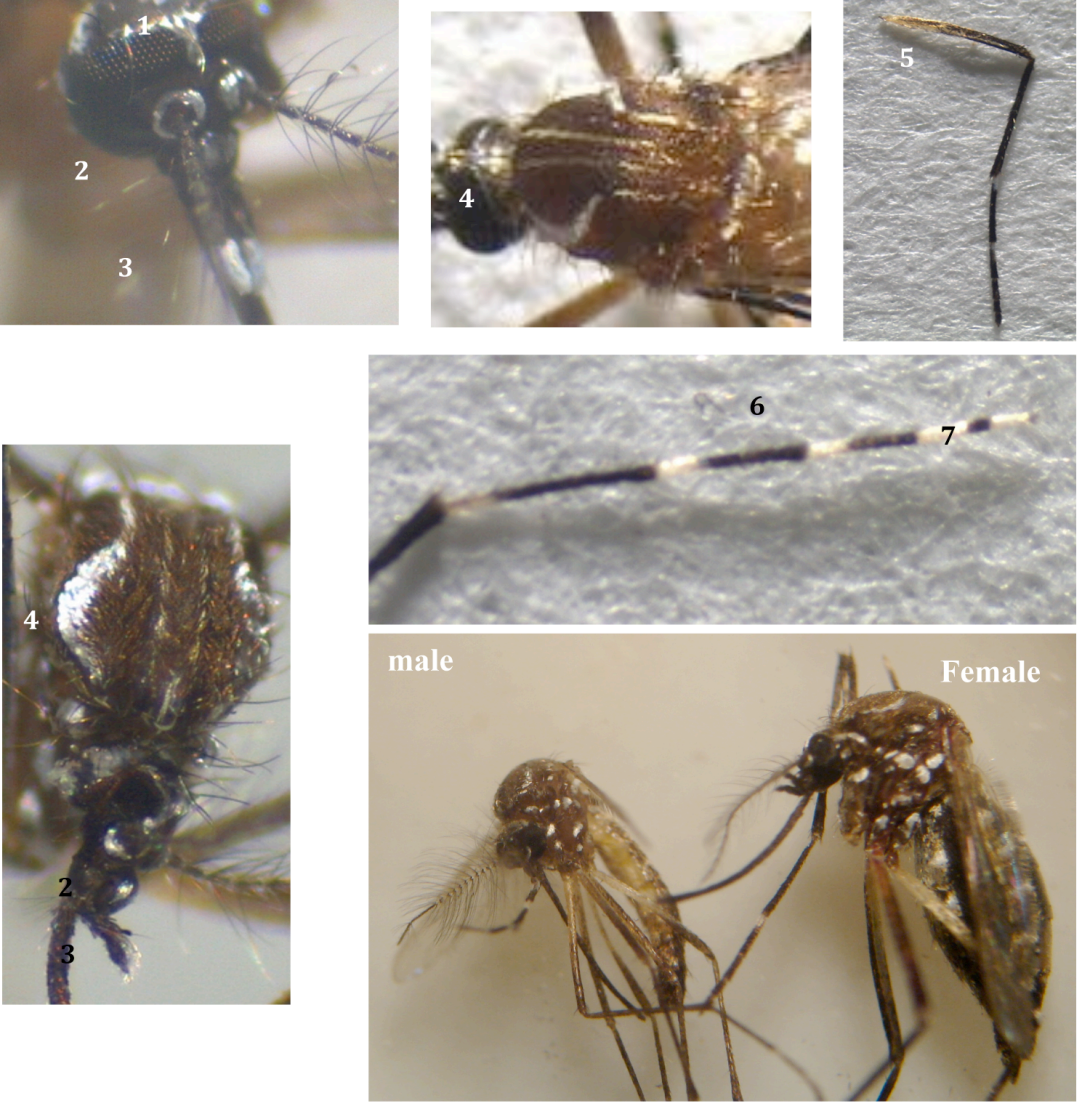
*Aedes (Stegomyia) aegypti* (L.). High quality figures are available online.

1. Silvery white scales on the head, especially on the vertex.
2. Clypeus with white silvery scales.
3. Palpi silvery white at tips.
4. Scutum with lyre-shaped silvery scale ornamentation.
5. Mid-femur with white longitudinal stripe from base to tip.
6. Hind tarsi with conspicuous white basal rings on 1–4 segments.
7. 5th segment of hind tarsi entirely white.

### *Aedes (Ochlerotatus) caspius* : ( Plate II )

**Plate II.**
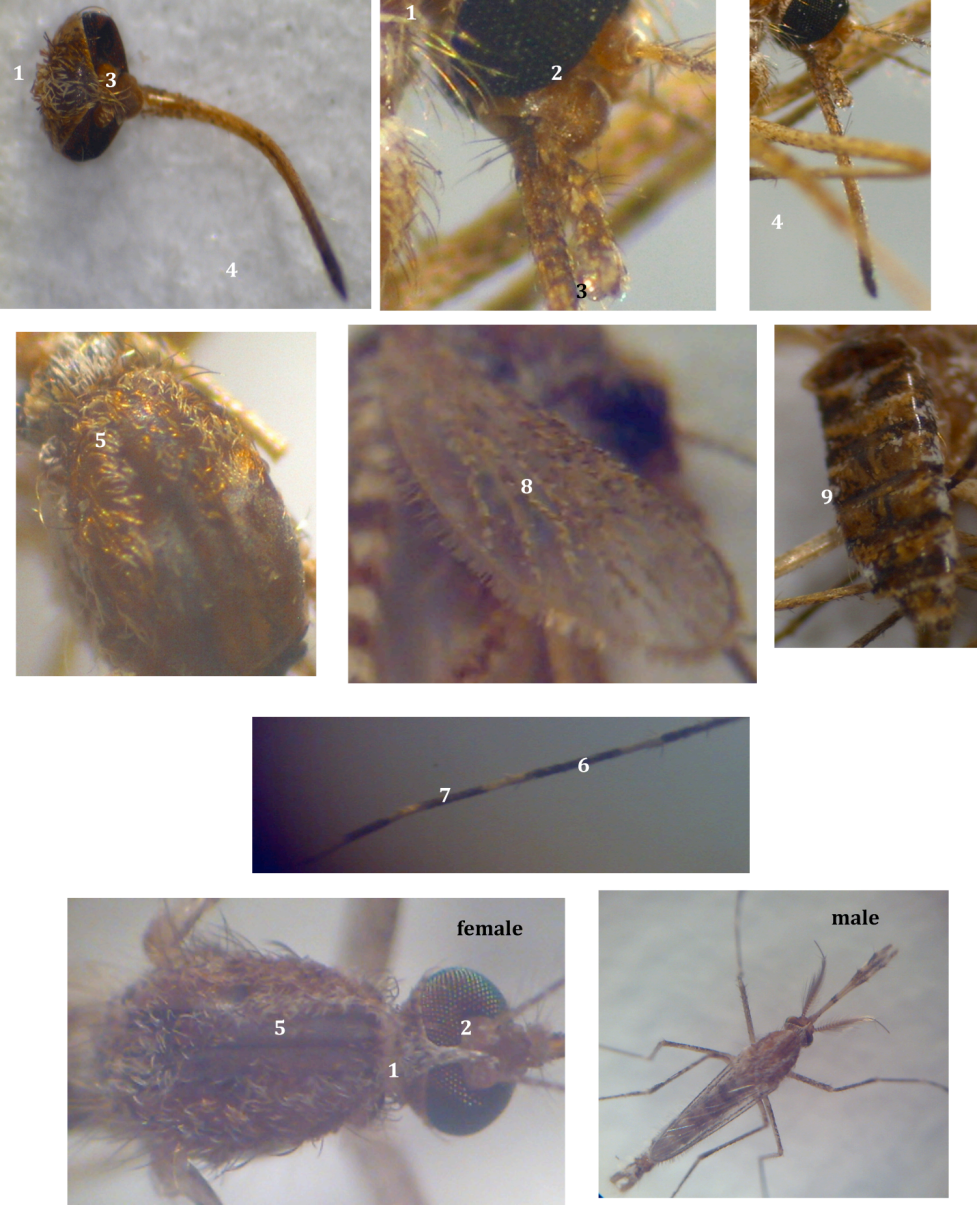
*Ochlertotatus* ( *Aedes* ) *caspius* (Pallas *)* . High quality figures are available online.

1. White upright scales on the vertex.
2. Clypeus pale brown.
3. Palpi with light and dark scales.
4. Proboscis dark at the tip but light in the middle and at the base.
5. Scutum golden or fawn colored scales with narrow dorsocentral stripes of white scales.
6. Tarsomeres with rings of pale scales.
7. Hind tarsomeres with both basal and apical pale rings.
8. Wings with white and dark scales. Base of costa mostly dark scales, vein 'R' with dark and pale white scales.
9. Abdominal terga with median pale stripes, sometimes entirely pale scales.

### *Aedes (Aedimorphus) vexans* var. *arabiensis* : ( Plate III )

**Plate III.**
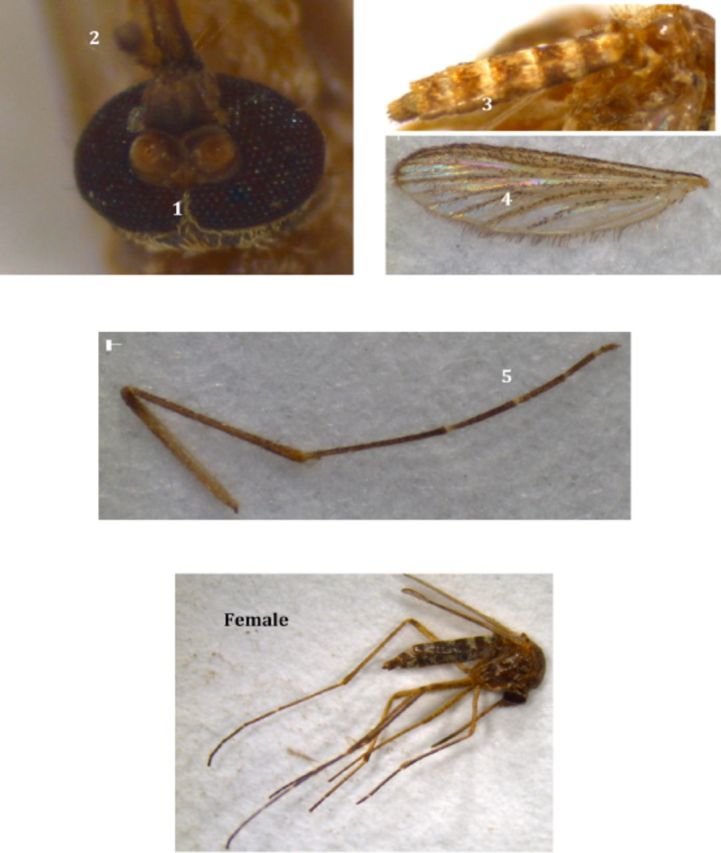
*Aedes (Aedimorphus) vexans* var . *arabiensis (* Meigen). High quality figures are available online.

1. Vertex with yellowish scales.
2. Palpi brown with pale scales at tips.
3. Plurae with many white or creamy scales.
4. Wings with dark scales.
5. Tarsi speckled with narrow basal pale rings.

### *Aedes (Aedimorphus) vittatus* : ( Plate IV )

**Plate IV.**
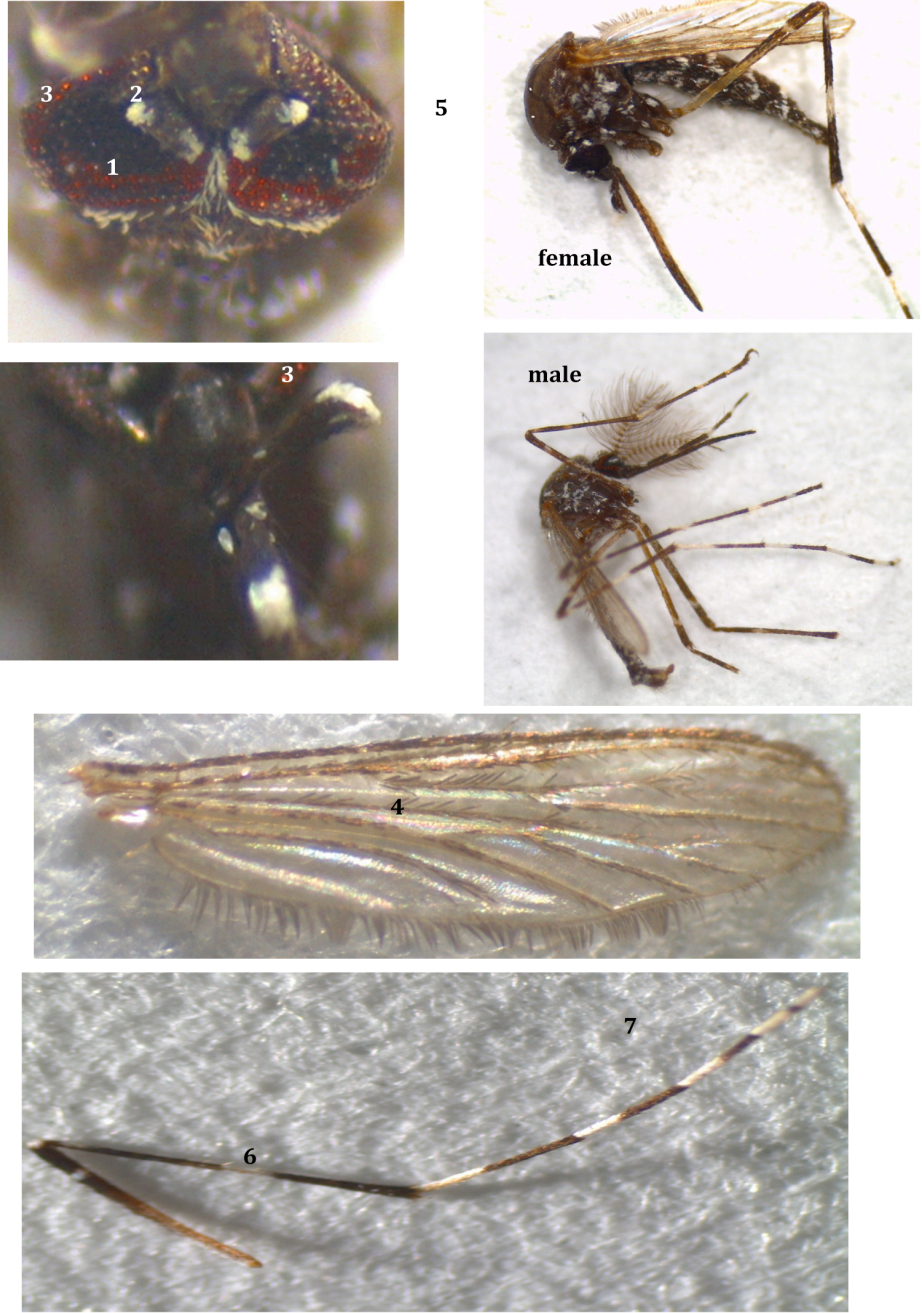
*Aedes (Aedimorphus) vittatus* (Bigot.). High quality figures are available online.

1. Silvery white scales on the narrow vertex.
2. Clypeus with white silvery scales.
3. Palpi with silvery white tips.
4. Wings with mainly narrow scales on all veins.
5. Scutum with three pairs of small round silvery white spots.
6. Tibiae are dark in color each with white spots and white sub basal white band.
7. Tarsomeres 1–4 with white bands; fifth tarsomere is fully white.

### Keys to the adult *Aedes* female mosquitoe species of Western Saudi Arabia

1. Clypeus with white scales, mid femur with an anterior white stripe from base to tip; scutum with lyre-shaped silvery scale…………………………... *aegypti* - Clypeus without scales (except *A. vit-tatus* ), scutum with other mark- mark- ings......................................................2

2. Scutum with three pairs of small, round, white spots; each femora with preapical white ring. Tibiae dark in color, each with white subbasal band........................................... *vittatus* - Otherwise marked……………….......3

3. Scutum with golden or fawn color scales with narrow darso-central stripes of white scales. Hind tarsomeres with both basal and apical rings, fifth hind tarsal segment pale, wings with white and dark scales......................................... *caspius* - Scutum with out darso-central white stripes..................................................4

4. Hind tarsi with pale rings confind to bases of segments, fifth hind tarsal segment wholly dark. Abdominal terga without median pale stripes.................. *vaxans* vr. *arabiensis*
